# Detection and characterization of latency stage of EBV and histopathological analysis of prostatic adenocarcinoma tissues

**DOI:** 10.1038/s41598-022-14511-4

**Published:** 2022-06-21

**Authors:** Khalid Ahmed, Alisalman Sheikh, Saira Fatima, Ghulam Haider, Kulsoom Ghias, Farhat Abbas, Nouman Mughal, Syed Hani Abidi

**Affiliations:** 1 Department of Biological and Biomedical Sciences, Aga Khan University, Karachi, Pakistan; 2 Section of Histopathology, Department of Pathology & Laboratory Medicine, Aga Khan University Hospital, Karachi, Pakistan; 3 Department of Surgery, Aga Khan University, Karachi, Pakistan; 4 Department of Biomedical Sciences, Nazarbayev University School of Medicine, Nur-Sultan, Kazakhstan

**Keywords:** Cancer, Microbiology, Oncology, Urology

## Abstract

The pathophysiology of prostate cancer involves both genetic and acquired factors, including pathogens, such as viruses. A limited number of studies have shown the presence of Epstein-Barr virus (EBV) in prostate cancer tissues. However, there is a dearth of data exploring EBV latency profile in prostate cancer, and the relationship of EBV with histopathological features of prostate cancer. In this study, prostate cancer and benign prostatic hyperplasia (BPH) samples were screened for the presence of EBV, followed by the characterization of the EBV latency profile and analysis of histopathological parameters in EBV-positive and EBV-negative groups. A conventional PCR strategy was employed using virus-specific primers to screen EBV in 99 formalin-fixed paraffin-embedded (FFPE) prostate cancer and 33 BPH samples received for histopathological analysis during the years 2019–2020. Subsequently, cDNA samples were used in a qPCR array to analyze the expression of EBV latency-associated genes to map the latency profile EBV maintains in the samples. Finally, statistical analyses were performed to determine the correlation between EBV and several histopathological features of the samples. EBV was detected in 39% of prostate cancer and 24% of BPH samples. The histopathological analysis of prostate cancer samples identified all samples as prostatic adenocarcinoma of acinar type, while statistical analyses revealed EBV-positive samples to exhibit significantly higher (p < 0.05) Gleason major and total Gleason scores as compared to EBV-negative samples. In the EBV-positive samples, variable expression patterns of latency-associated genes were observed, where most of the samples exhibited EBV latency II/III-like profiles in prostate cancer, while latency-II-like profiles in BPH samples. This study suggests a high prevalence of EBV in prostate samples, where EBV exhibited latency II/III-like profiles. Furthermore, EBV-positive samples exhibited a higher Gleason score suggesting a possible link between EBV and the onset/progression of prostate cancers. However, future functional studies are required to understand the role of the EBV gene expression profile in the onset/progression of prostate cancer.

## Introduction

Globally, prostate cancer is the most frequently diagnosed cancer in men^1^. The pathophysiology of prostate cancer involves both genetic and acquired factors. Despite being extensively studied, several molecular events responsible for the de novo emergence and progression of neoplastic change in the prostate gland remain unclear^2^. Recent studies have shown an association between certain viral infections and the onset/progression of prostate carcinoma. The most common infections associated with prostate carcinoma are human papillomavirus (HPV), human herpesvirus type 8 (HHV8), Epstein-Barr virus (EBV), and human herpes simplex virus type 2 (HHV2), etc.^3,4^. However, little is known about the molecular changes that these viral agents may induce in the prostate epithelial cells that might lead to the initiation or progression of prostate cancer. Some studies have demonstrated the ability of HHV8 to modulate JAK/STAT3, VEGF, and NF-κB pathways as well as downregulate androgen receptor expression to promote prostate cancer progression^3,5^.

Similar to HHV-8, EBV is a known human oncovirus, which has been labeled as Group I carcinogenic risk factor to humans based on causality for nasopharyngeal carcinoma, gastric carcinoma, Burkitt’s lymphoma, non-Hodgkin’s lymphoma (immunosuppression related), and Hodgkin’s lymphoma^6^. EBV has predominantly been shown to maintain type II latency profiles in the nasopharyngeal carcinoma^7,8^, while in the EBV-associated gastric carcinoma and Burkitt’s lymphoma, EBV predominantly maintains the latency I profile^9^. The immunosuppressive patients, however, who develop EBV-associated lymphomas tend to have a latency III profile. The expression of EBV latency-associated genes has been associated with invasion, cell proliferation, and inhibition of apoptosis^10^.

EBV has been reported in prostate cancer epithelial cells^11–13^. However, there is a dearth of data exploring virological features, such as the latency profile that EBV maintains in prostate cancer samples or the relationship between EBV and histopathological features of cancer.

In this study, we attempted to fill this gap by performing PCR-based screening of prostate cancer samples to detect EBV, followed by the analysis of the expression of EBV latency-associated genes to characterize the EBV latency stage profile in prostate carcinoma biopsy samples. Finally, we analyzed the relationship between EBV and several histopathological features of prostate cancer.

## Materials and methods

### Sample collection

The sample size for this study was calculated considering the incidence of prostate cancer in Pakistan to be 5.3% per 100,000 people, with a marginal error, and a confidence interval of 5% and 95%, respectively^14,15^. Using the above parameters, the sample size was calculated to be 73. However, we were able to procure a total of 99 Formalin-fixed, paraffin-embedded (FFPE) tissue blocks from the year 2019, with a confirmed diagnosis of prostate cancer by a trained histopathologist. All, but three, prostate carcinoma specimens were oncologically treatment naïve (n = 96), which were obtained after transurethral resection of the (TURP) (n = 68), or sextant needle biopsy (n = 25), and were sent to the laboratory for their first histopathological biopsy assessment. However, three samples were obtained after radical prostatectomy, and these patients may have received oncological treatment. In addition, a total of 33 FFPE tissue blocks from the year 2020, with a confirmed diagnosis of benign prostatic hyperplasia (BPH) were also included to provide a comparative analysis of the presence of EBV in both BPH and prostate carcinoma samples, along with the characterization of the expression of EBV latency-associated genes. All the BPH samples which were included in this study exhibited glandular and stromal hyperplasia. The prostate carcinoma samples and BPH samples were not consecutive but were collected independently of each other by purposive and convenience sampling, respectively. The samples/blocks were obtained from the Department of Pathology and Laboratory Medicine at the Aga Khan University (AKU), Karachi after obtaining informed consent from all subjects. The study was approved by the Aga Khan University Ethics Review Committee (AKU-ERC #: 2021-1460-18525). All methods were performed as per the relevant guidelines and regulations.

### Histopathological analysis of prostate cancer biopsy samples

The FFPE tissue blocks were used to prepare slides for hematoxylin and eosin staining using standard protocol^16^. The tumors were graded using the International Society of Urological Pathology (ISUP) 2014 / WHO 2016 prostate cancer grade group system^17^. The specimens were graded by documenting histopathological parameters, such as Gleason major, minor and total scores, and prognostic grade groups in the samples based on the standard protocol^17^. Gleason grade groups were assigned as per the total Gleason scores, viz. Gleason grade group 5 (Gleason score 9–10), Gleason grade group 4 (Gleason score 8), Gleason grade group 3 (Gleason score 4 + 3 = 7), Gleason grade group 2 (Gleason score 3 + 4 = 7), and Gleason grade group 1(Gleason score 6). To compare the difference in the mean Gleason scores (major, minor, and total) between EBV-positive and EBV-negative prostate cancer samples, independent samples t-test was used. Similarly, the Pearson Chi-Square test was applied to determine the association of intratumoral lymphocytes, stromal lymphocytes, and benign tissue lymphocytic infiltration with EBV status (positive or -negative), while the Spearman correlation test was applied to study the relationship between different histopathological parameters and EBV status. For all the statistical tests used in this study, a p < 0.05 was considered statistically significant. IBM-SPSS version 23.0 was used to analyze the data.

Given the significance of EBV-associated lymphocytic infiltration in other EBV-associated epithelial cancers, such as nasopharyngeal carcinoma, and its corresponding association with the prognosis^18^, we aimed to analyze the status of lymphocytic infiltration in EBV-positive and EBV-negative prostate carcinoma specimens. A modified version of the set criteria for solid tumors by the International Immuno-Oncology Biomarker Working Group was used^19^, where all mononuclear cells were scored and were characterized as either intratumoral lymphocytic infiltration—where the lymphocytic cells were in contact with tumor cells, or stromal lymphocytic infiltration—where the lymphocytes were not in direct contact with tumor cells. The intratumoral and stromal tissue lymphocytic infiltration was independently verified by a trained histopathologist. To characterize the number of the infiltrating lymphocytes in the prostate cancer samples on light microscopy, the prostate cancer Hematoxylin and Eosin-stained slides were scanned for the presence of lymphocytes, and the cells were counted within tumor cells, stromal part and in the adjacent benign tissue. Depending on the number of lymphocytes present, the observations were divided into the following four categories: zero (no lymphocytes seen), + 1 (1–15 lymphocytes seen), + 2 (16–25 lymphocytes seen), + 3 (> 25 lymphocytes seen), respectively.

### DNA extraction from prostate carcinoma and benign prostatic hyperplasia FFPE tissue blocks

For each given sample, four 10 μm thick FFPE sections were cut using a microtome and were stored at room temperature into two autoclaved 1.5 ml microcentrifuge tubes each for RNA and DNA extractions until further use. In the first step, the sample was deparaffinized. For this purpose, each sample was washed with 1000 μl of xylene and mixed by vortexing for 30 s, followed by incubation for 10 min on a shaker at room temperature. After incubation, the tubes were centrifuged at 15,000 rpm for 2 min, and subsequently, the xylene was removed. This step was repeated twice until all the paraffin was replaced by xylene. In the next step, xylene was removed from the tissues using 100% ethanol. For this purpose, 1.5 ml of 100% ethanol was added to each sample and mixed using a vortex for 30–60 s. After this, the tubes were left on the shaker for 5 min and then centrifuged at 15,000 rpm for 2 min followed by the removal of the liquid phase. This step was repeated twice until all the xylene was removed. Finally, the samples were gradually rehydrated using different concentrations of ethanol (95% and 70% respectively). For this purpose, 1.5 ml of 95% ethanol was added to the dehydrated samples and mixed using vortexing for 30 s, after which they were kept on a shaker for 10 min and then centrifuged at 15,000 rpm for 2 min. This step was repeated once using each concentration, and the final step was performed using deionized water. Finally, DNA was extracted from each sample using DNeasy Blood & Tissue kit (Qiagen, USA), following the manufacturer's instructions. The DNA was stored at – 80 °C until further use.

### Conventional PCR for the detection of EBV in prostate cancer and benign prostatic hyperplasia samples

To detect the presence of EBV in the prostate cancer samples (n = 99), conventional PCR was employed using the EBNA-2 gene primers, since EBNA-2 is constitutively expressed in EBV-infected cells^20^. For PCR reaction, 100–150 ng of DNA template was combined with the 4 μl of BesTaq master mix (2X) (ABM, Canada, Cat. no. G464), 1 pM of custom-made forward and reverse primers (Table 1) (Macrogen, USA) and nuclease-free water to a final volume of upto 10μl. The above reaction was used in PCR with the following cycling conditions: initial denaturation for 10 min at 95 °C, followed by 36 cycles of denaturation for 15 s at 95 °C, annealing for 1 min at 60 °C, and an extension for 30 s each at 72 °C, followed by the final extension for 1 min at 72 °C. The amplicons from the reaction were analyzed on 1.8% agarose gel against a 50-bp ladder (Promega, USA) using the ChemiDoc imaging system (Bio-Rad Laboratories, USA). The amplicons showing bands at 96 bps were considered positive for EBNA-2.

**Table 1:** EBV latency-associated genes along with sequences of the forward and reverse primers.

Genes	Sequence (5’–3’)
*EBNA-1*	Fwd TACAGGACCTGGAAATGGCC Rev TCTTTGAGGTCCACTGCCG
*EBNA-2*	Fwd GCTTAGCCAGTAACCCAGCACT Rev TGCTTAGAAGGTTGTTGGCATG
*EBNA-3A*	Fwd CCCCTTAACTCAACCCATTAACC Rev CGGCCCCTCCATTGGT
*EBNA-3B*	Fwd TGCCGCTGCAAGAGAGG Rev AGGTCCGATTGCAACATGGA
*LMP-1*	Fwd CAGTCAGGCAAGCCTATGA Rev CTGGTTCCGGTGGAGATGA
*LMP-2*	Fwd GGTTCTCCTGATTTGCTCTTCGT Rev CGCGGAGGCTAGCAACA
*LMP-2A*	Fwd TCCCTAGAAATGGTGCCAATG Rev GAAGAGCCAGAAGCAGATGGA
*EBER-1*	Fwd TGCTAGGGAGGAGACGTGTGT Rev TGACCGAAGACGGCAGAAAG
*EBER-2*	Fwd AACGCTCAGTGCGGTGCTA Rev GAATCCTGACTTGCAAATGCTCTA
*BZLF-1*	Fwd AAATTTAAGAGATCCTCGTGTAAAACATC Rev CGCCTCCTGTTGAAGCAGAT
*BHRF-1*	Fwd GGCTTACCTCGGTTCCCTCTTA Rev TCCCGTATACACAGGGCTAACAGT
*EBNA-3C*	Fwd TACGCCCCATTCCAACAAG Rev CCCACGGCCATGCTATCTT
*LMP-2B*	Fwd GTAATCTGCACAAAGAGGCGC Rev AAAGCACGGCCTCCCG
*EBNA-LP*	Fwd AGAGACCACTTTGAGCCAC Rev TCAGGCAAAACTTTACACCAC

*Fwd* forward primer, *Rev* reverse primer.

### Extraction of RNA by TRIzol-chloroform method and cDNA synthesis

RNA was extracted from the tissues using the TRIzol-chloroform method^21^. Briefly, following the tissue digestion, 700 μl of TRIzol reagent (Invitrogen, Thermo Fisher Scientific, Inc.), was added to each sample and the samples were allowed to incubate on ice for 5 min to allow the disassociation of nucleoprotein complexes. After the incubation, 200 μl of chloroform was added to each tube, and the contents were mixed vigorously, followed by another incubation at 4 °C for 10–15 min, and centrifugation for 5 min at 12,000 rpm, to allow the phase separation in the mixture. Following this centrifugation, the upper aqueous phase, where RNA is concentrated, was transferred to a fresh autoclaved 1.5 ml microcentrifuge tube without disturbing the interphase and chilled 1000 μl isopropyl alcohol was added and incubated for 10 min at room temperature to chelate the RNA from the aqueous phase. Following the incubation, the tubes were centrifuged for 10 min at 12,000 rpm to obtain a white pellet of pure RNA at the bottom of the tube. The pellet was washed with 1000 μl of 70% ethanol and left to air dry. The pellet, containing RNA, was finally resuspended in 50 μl of nuclease-free water. The RNA was stored at − 80 °C until further use.

To remove the genomic DNA contamination in the RNA samples, and before cDNA synthesis, total RNA was treated with DNase I. For this purpose, 1 μg of the total RNA template was combined in a 0.2 ml tube with 1 μl of (10X) reaction buffer containing MgCl_2,_ 1 μl of DNase-I, RNase-free 1U/1 μl (Thermo Fisher Scientific, Cat. No. EN0521), and suitable volume of nuclease-free water for a final volume of up to 10 μl. The prepared reaction was incubated for 30 min at 37 °C in the Master cycler X50a (Eppendorf, Germany). To prevent the hydrolysis of RNA after the DNase-I treatment, 1 μl of 50 mM EDTA was added and samples were allowed to incubate for 10 min at 65 °C. The DNase-I treated total RNA, from the above step, was converted to cDNA using the OneScript plus cDNA synthesis kit (ABM, Canada. Cat. No. G236) following the manufacturer's instructions and stored at − 20 °C till further use.

### Latency mapping of EBV in prostate cancer and benign prostatic hyperplasia tissue samples using quantitative Polymerase Chain Reaction (qPCR)

Following the identification of EBV-positive prostate cancer tissues, a quantitative real-time PCR was employed to analyze the expression of EBV latency-associated genes (*EBNA-3B, EBNA-3A, EBNA-2, EBNA-1, LMP-2A, LMP-2, LMP-1, EBNA-LP, EBNA-3C, EBNA-2B, EBER-2, EBER-1, BZLF-1, and BHRF-1*) and determine the EBV-latency profile in prostate cancer samples. For this purpose, 2 μl of cDNA sample was combined with a mixture containing 4 μl of BlasTaq (2X) qPCR master mix (ABM, Canada, Cat. No. G891), forward and reverse gene-specific primers (Table 1) (Macrogen, USA) and nuclease-free water to a final reaction volume of up to 10 μl in a 0.2 ml tubes (Bio-Rad Laboratories, USA. Cat. No. TLS0851). The prepared reactions were subjected to the following thermal cycling conditions using Bio-Rad 1000 thermal cycler CFX96 (Bio-Rad laboratories, USA): initial denaturation for 10 min at 95 °C, followed by 40 cycles for denaturation for 15 s at 95 °C, annealing for 1 min at 45 °C, and a cyclic extension for 30 s at 72 °C). A melt curve analysis was set up between 55 °C and 95 °C with an increment of 0.5 °C every 5 s to plot the specificity of the products. Each sample was run in duplicates, while non-template controls were supplied with an additional 2 μl of nuclease-free water instead of a cDNA template.

## Results

### Detection of EBV in prostate cancer samples and correlation of histopathological parameters

**Figure 1:**
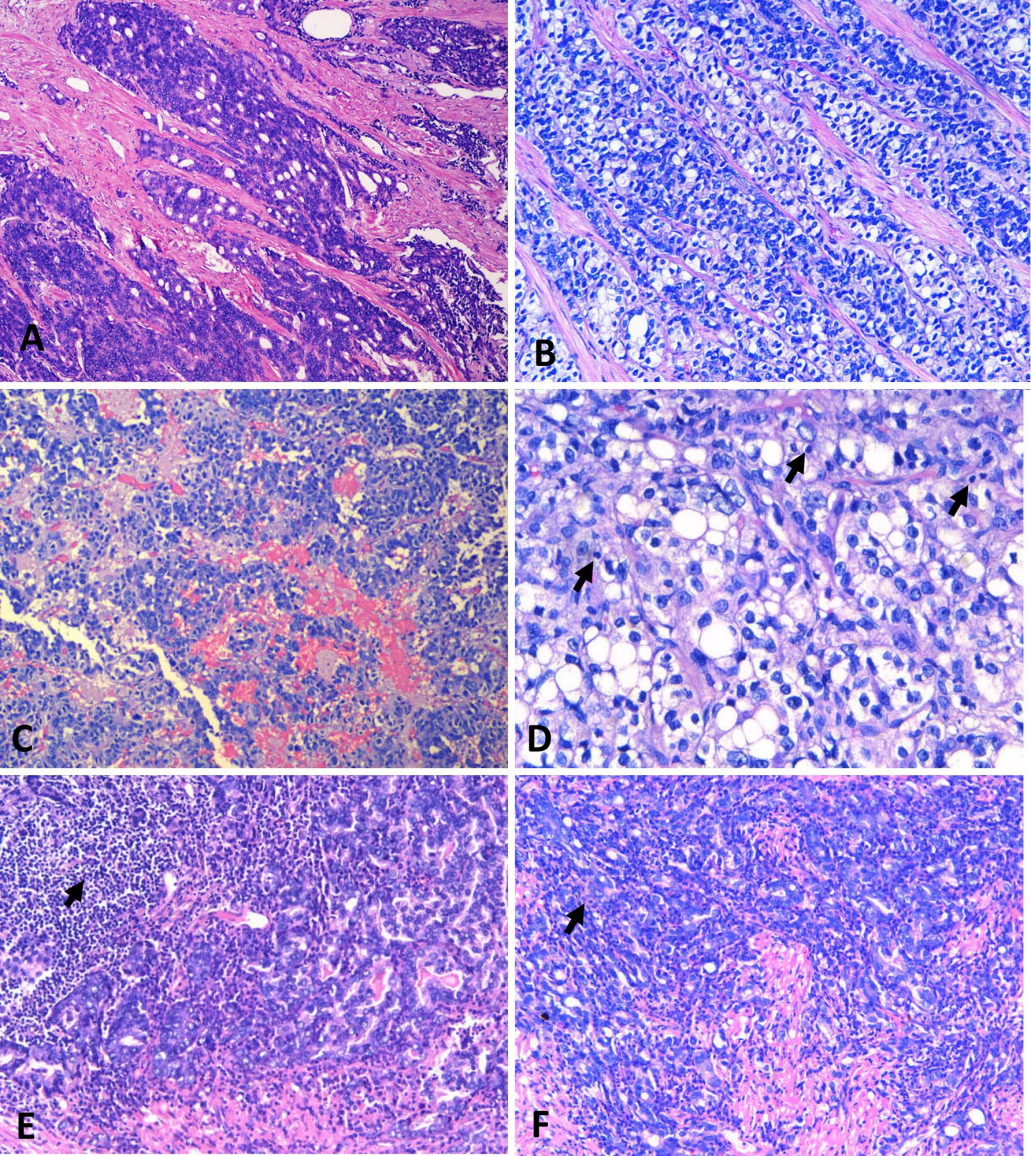
Representative histopathological images of EBV-positive and EBV-negative prostate adenocarcinoma of the acinar type showing the Gleason pattern where higher Gleason scores are found in EBV-positive prostate carcinoma tissues as compared to EBV-negative prostate carcinoma tissues. The histopathological images show: (**A)** EBV-positive prostate adenocarcinoma, Gleason score of 9 (4 + 5), with a prognostic grade group 5 (H&E; original magnification: × 100). (**B)** EBV-negative prostate adenocarcinoma, Gleason score of 8 (4 + 4), with a prognostic grade group 4 (H&E; original magnification: 200X). (**C)** EBV-positive prostate adenocarcinoma, Gleason score of 9 (4 + 5), with a prognostic grade group 5 (H&E; original magnification: × 100). **(D)** EBV-positive prostatic adenocarcinoma, Gleason score of 9 (4 + 5) showing the intratumoral lymphocytes (ITL) infiltration in the tumor marked with black arrows (H&E; 400X). (**E)** EBV-negative prostate adenocarcinoma, Gleason score of 9 (4 + 5), with a prognostic grade group 5 with stromal lymphocytic infiltration (black arrows arrow) (H&E; original magnification: × 200) (**F)** EBV-negative prostate adenocarcinoma, Gleason score of 9 (4 + 5), with a prognostic grade group 5 with the intratumoral lymphocytic infiltration. (H&E; original magnification: × 200).

Out of 99 FFPE prostate cancer tissue, 39.39% were found to be positive for EBV. All 99 samples were identified as prostatic adenocarcinoma of acinar type. In the EBV-positive group, all samples, except three, were diagnosed as acinar adenocarcinoma. The three samples showed the following variants of acinar adenocarcinoma in the EBV-positive group: one was a microcystic variant, one was a foamy gland variant, and one was a signet ring-like cell variant of acinar adenocarcinoma. In the EBV-negative group, all, but seven, samples were diagnosed as acinar adenocarcinoma. In seven samples the variants were identified as followed: 3 were foamy gland variants, 3 were signet ring-like cell variants, and 1 was of the atrophic variant of acinar adenocarcinoma. Analysis of histopathological parameters in EBV-positive and -negative samples showed the Gleason major scores (4.12 ± 0.64 vs 3.84 ± 0.58) and total Gleason scores (8.24 ± 1.22 vs 7.75 ± 1.03) to be significantly higher (p < 0.05) in EBV-positive and EBV-negative samples, respectively. These observations were further supported by Spearman’s rho test, where a weak positive correlation (R-value: 0.206; p = 0.041*)* was observed between EBV positive status and Gleason major and total scores (Table 2). The association with additional histopathological features, such as prognostic grade group, intratumoral lymphocytes, and stromal lymphocytes were not significantly different between the EBV-positive and -negative samples. Notably, the infiltration of lymphocytes in benign tissue adjacent to the tumor tissue was found to be zero in 15.9% of EBV-negative and 3.8% of EBV-positive samples, while 1 + in 52.3% of EBV-negative and 76.9% of EBV-positive samples (Fig. 1).

**Table 2:** Comparison of mean Gleason scores of EBV-positive and EBV-negative groups using independent samples t-test.

Histopathological parameter(s)	EBV-positive & EBV-negative samples	N	Mean	SD	p-value
Gleason score major	Negative	66	3.84	0.58	0.038*
	Positive	33	4.12	0.64	
Gleason score minor	Negative	66	3.90	0.62	0.18
	Positive	33	4.12	0.78	
Gleason score total	Negative	66	7.75	1.03	0.042*
	Positive	33	8.24	1.22	
Prognostic grade group	Negative	63	3.68	1.35	0.71
	Positive	36	3.58	1.25	

The table shows the mean score comparison of Gleason major, Gleason minor, and total Gleason scores in between positive and negative samples. The histopathological analysis of tumor tissues was done as per the International Society of Urological Pathology (ISUP) 2014 and later ratified by WHO 2016 as the prostate cancer grade group system.

*p < 0.05 was considered significant using an independent sample t-test.

### Detection of EBV and latency mapping in prostate cancer biopsies

The expression of EBV latency-associated genes was analyzed to determine the EBV latency profiles in the prostate cancer samples (Table 3). The analysis showed variable expression patterns of latency-associated genes in EBV-positive samples, where most of the samples exhibited non-classical EBV latency II/III-like profiles (Table 3). The Ct values for each latency-associated gene are given in Supplementary Table S1.

**Table 3:** Characterization of EBV latency profile in EBV-positive prostate carcinoma tissues.

Sample ID	*EBNA-LP*	*EBNA-1*	*EBNA-2*	*EBNA-3A*	*EBNA-3B*	*EBNA-3C*	*LMP-1*	*LMP-2*	*LMP-2A*	*LMP-2B*	*EBER-1*	*EBER-2*	*BHRF-1*	*BZLF-1*
Prostate_RNA_7	+	+	+	+	+	−	+	+	+	−	+	+	+	+
Prostate_RNA_12	+	+	+	+	+	−	+	+	+	−	+	+	+	+
Prostate_RNA_14	+	+	+	−		−	+	+	+	+	+	+	+	+
Prostate_RNA_16	−	+	+	−	+	−	+	+	+	+	+	−	−	−
Prostate_RNA_17	+	+	+	+	−	−	+	+	+	−	+	+	+	+
Prostate_RNA_19	−	+	+	−	−	−	−	+	−	+	+	−	−	−
Prostate_RNA_21	+	+	+	+	+	−	+	−	+	+	+	−	+	−
Prostate_RNA_22	−	+	+	−	−	−	−	−	+	+	+	−	−	+
Prostate_RNA_24	+	+	+	−	+	+	+	+	+	+	+	+	+	+
Prostate_RNA_27	+	+	+	+	+	+	+	+	+	+	+	+	+	+
Prostate_RNA_28	−	+	+	+	+	−	+	−	+	−	+	−	−	−
Prostate_RNA_34	+	+	+	+	+	−	+	−	+	−	+	+	+	−
Prostate_RNA_38	+	+	+	+	−	+	+	+	+	+	+	−	+	+
Prostate_RNA_40	−	+	+	−	−	−	+	−	+	+	+	+	+	+
Prostate_RNA_41	−	+	+	−	+	−	+	+	+	+	+	+	+	+
Prostate_RNA_42	−	−	+	−	+	−	+	+	+	+	+	+	+	+
Prostate_RNA_43	+	+	+	−	−	−	+	+	+	+	+	−	+	+
Prostate_RNA_51	−	+	+	−	+	−	+	−	+	+	+	+	−	+
Prostate_RNA_52	+	+	+	+	+	+	+	+	+	+	+	+	+	+
Prostate_RNA_53	−	+	+	−	+	−	+	+	+	+	+	+	+	+
Prostate_RNA_54	−	+	+	−	+	−	+	+	+	+	+	−	−	+
Prostate_RNA_59	−	+	+	−	−	−	+	−	+	+	+	−	−	+
Prostate_RNA_63	+	+	+	+	+	+	+	+	+	+	+	+	+	+
Prostate_RNA_64	−	+	+	−	+	−	+	+	+	+	+	−	−	+
Prostate_RNA_ 65	+	+	+	−	+	−	+	−	−	−	+	−	−	+
Prostate_RNA_66	+	+	+	−	−	+	+	−	+	−	−	+	−	+
Prostate_RNA_69	+	+	+	+	+	−	+	+	+	−	+	+	+	+
Prostate_RNA_72	+	+	+	−	−	−	+	−	+	−	+	−	+	+
Prostate_RNA_77	+	+	+	+	+	+	+	+	+	+	+	+	+	+
Prostate_RNA_79	+	+	+	+	+	−	+	−	+	+	+	+	−	+
Prostate_RNA_82	−	+	+	−	−	−	+	−	+	+	−	+	−	+
Prostate_RNA_86	+	+	+	+	+	+	+	+	+	+	−	+	+	+
Prostate_RNA_89	+	+	+	+	+	+	+	+	+	+	+	+	+	+
Prostate_RNA_90	−	+	+	+	+	−	+	−	+	−	+	+	+	+
Prostate_RNA_99	−	+	+	−	+	−	+	+	+	+	+	+	−	+
Prostate_RNA_100	−	+	+	−	+	−	+	+	+	+	+	+	+	+
Prostate_RNA_102	+	+	−	+	−	−	+	+	+	+	+	+	+	+
Prostate_RNA_106	+	+	+	+	−	+	+	+	+	+	+	−	+	+
Prostate_RNA_109	−	+	−	−	−	−	+	−	−	−	+	−	+	+

The latency profile was mapped based on the expression of the following latency-associated genes: *EBNA-3B, EBNA-3A, EBNA-2, EBNA-1, LMP-2A, LMP-2, LMP-1, EBNA-LP, EBNA-3C, EBNA-2B, EBER-2, EBER-1, BZLF-1,* and *BHRF-1.* The + and − signs show the presence and absence of gene expression, respectively.

### Detection of EBV and Latency mapping in benign prostatic hyperplasia (BPH) biopsies

We found 24.24% (8 out of 33 of the samples) of BPH biopsy samples to be EBV positive. In the next step, we analyzed the expression of EBV latency-associated genes (Table 1) to determine the EBV latency profiles in the benign prostatic hyperplasia samples. The analysis showed variable expression patterns of latency-associated genes in EBV-positive samples, where all of the samples except one, that exhibited a latency-III-like profile, exhibited a latency II-like profile (Table 4). The Ct values for each latency-associated gene are given in Supplementary Table 2.

**Table 4:** Characterization of EBV latency profile in EBV-positive BPH samples.

Sample ID	*EBNA-LP*	*EBNA-1*	*EBNA-2*	*EBNA-3A*	*EBNA-3B*	*EBNA-3C*	*LMP-1*	*LMP-2*	*LMP-2A*	*LMP-2B*	*EBER-1*	*EBER-2*	*BHRF-1*	*BZLF-1*
BPH_03	+	+	+	−	−	−	+	+	+	+	+	+	−	+
BPH_05	−	+	+	−	−	−	+	+	+	+	+	+	−	+
BPH_14	+	+	+	−	−	−	+	+	+	+	+	+	−	+
BPH_16	+	+	+	+	+	+	−	+	+	+	+	+	+	+
BPH_20	−	+	+	−	−	−	+	+	+	+	+	+	−	+
BPH_21	+	+	+	−	−	−	+	+	+	+	+	+	+	+
BPH_23	+	+	+	−	−	−	+	+	+	+	+	+	−	+
BPH_28	+	+	+	−	−	−	+	+	+	+	+	+	−	+

The latency profile was mapped based on the expression of the following latency-associated genes: *EBNA-3B, EBNA-3A, EBNA-2, EBNA-1, LMP-2A, LMP-2, LMP-1, EBNA-LP, EBNA-3C, EBNA-2B, EBER-2, EBER-1, BZLF-1,* and *BHRF-1.* The + and − signs show the presence and absence of gene expression, respectively.

## Discussion

In this study, we have screened prostate cancer samples for the presence of EBV, followed by the characterization of the EBV latency profile in prostate carcinoma samples and analysis of histopathological parameters in EBV-positive and EBV-negative groups.

Of the prostatic adenocarcinoma of acinar subtype samples, 39.39% of the samples were EBV positive. Our study is consistent with earlier studies, where EBV was identified in 40% of prostate cancer tissues in Australian males based on in situ PCR technique^11^, and 37% of prostatic adenocarcinoma samples in the US based on immunohistochemistry^22^. In an Iranian study, EBV was found to be present in 49.3% of the prostate cancer samples^23^. Similarly, an Australian study found a comparable (40%) distribution of EBV in both prostate carcinoma samples as well as normal prostate tissue^11^. Although, studies have reported the detection of EBV in prostate specimens, however, the role of EBV in the onset and progression of prostate cancer remains a moot point. For example, a Swedish study reported no association between the presence of viruses, including EBV, inflammatory prostate status, and thus the development of cancerous lesions^24^. However, the scope of the above-mentioned studies has been limited to only the detection of EBV in both prostate cancer and normal prostate tissues, and none of them have undertaken the analysis of latency profiling of EBV in the prostate cancer samples.

The screening of the BPH samples showed the presence of EBV in 24.24% of the samples, where EBV predominantly exhibited a type II-like latency profile. Our reported EBV positivity percentage in BPH samples is higher than all previously reported studies. An Iraqi study found an EBV-positive percentage of 10% in BPH samples based on immunohistochemical analysis of EBV-EBER proteins^25^. Another Iraqi study reported 5% and 25% of EBV-positive status in benign prostatic hyperplasia samples and prostate carcinoma, respectively^26^. Further studies need to be carried out to establish the difference between EBV infection and latency in benign prostatic hyperplasia and prostate adenocarcinoma.

In EBV-positive epithelial cancers, the expression of EBV latency genes has been reported to be found in almost all cancerous cells^27^, thereby, showing a strong association between EBV and EBV-positive epithelial cancers. Our findings show that EBV exhibits non-classical EBV latency II/III-like profiles in adenocarcinoma of prostate cancer based on the expression of *EBNA-3B, EBNA-3A, EBNA-2, EBNA-1, LMP-2A, LMP-2, LMP-1, EBNA-LP, EBNA-3C, EBNA-2B, EBER-2, EBER-1, BZLF-1, and BHRF-1* (Table 3). EBV infection with subsequent expression of EBV latency genes products might enhance the survival of premalignant cells along with the conferred antiapoptotic properties to epithelial cells which are in the premalignant state, thereby, increasing the chances of harboring genetic alteration or mutations^28^. At the same time, the genetic changes, such as inflammation in stromal portions of tissues, may further result in modulation of EBV latency gene expression which may alter the growth properties of the tissue and might preselect and pave the pathway towards carcinogenesis^29^. These findings are comparable to earlier reports that have shown that EBV maintains a type II latency profile in EBV-associated epithelial cancers, such as EBV-associated gastric carcinoma (EBVaGC) and nasopharyngeal carcinoma^28^. Recent studies have reported that LMP2A and LMP1 play a role in epithelial-to-mesenchymal transition in nasopharyngeal carcinoma, thereby, playing a critical role in the onset of cancer^30^, and IHC expression of LMP1 of EBV has been recently reported in the prostate carcinoma samples^31^. Although *BZLF1* is an intermediate early gene, few studies have reported its crucial role in establishing and regulating the EBV latency in the host cells^32,33^. BZLF1 has also shown to be expressed during early EBV infection without inducing EBV lytic cycle with a role in the pre-latent phase of EBV infection^34^.

Histopathological analysis of EBV-positive and -negative tissues showed a significant difference in EBV-positive and EBV-negative prostate adenocarcinoma samples, where EBV-positive samples exhibited a higher Gleason score when the comparison of means of Gleason scores was made between two groups (Table 2). Tumor grading based on the Gleason score is a strong predictor of the prognosis, and higher Gleason scores are associated with a worse prognosis^35^. We found higher mean Gleason scores in EBV-positive prostate carcinoma samples as compared to EBV-negative prostate carcinoma samples, which may suggest a link between EBV infection and aggressive form of prostate carcinoma. Although, prior to this report, nothing is known about the association of EBV-positive prostate carcinoma with the Gleason scores. However, a study has reported a significant association between HPV infection in prostate carcinoma and corresponding high Gleason scores^36^. However, further studies are required to understand the putative mechanisms by which EBV leads to aggressive forms of prostate carcinoma.

We identify certain limitations of this study. Firstly, a limited number of BPH samples were included to perform a comparison in EBV status and expression of latency genes between cancerous and non-cancerous prostate tissues. Secondly, due to limited resources, we could not characterize sub-populations of infiltrating lymphocytes in EBV-positive and EBV-negative samples. This aspect can be studied in future studies using a cluster of differentiation markers for the lymphocytes to understand the differences in the immune response of EBV-positive samples versus EBV-negative samples.

In summary, the presence of EBV in prostate cancer tissues, maintenance of type II/III profiles, and association with a high Gleason score might suggest a link between EBV infection and initiation and/or promotion of prostate cancer. However, further studies are required to fully understand the how gene expression profile of EBV contributes to prostate cancer progression.

## Supplementary Information

Supplementary Tables.

## Data Availability

All data generated or analyzed during this study are included in this published article (and its supplementary information files).
