# Supplementary material for: Detection and characterization of latency stage of EBV and histopathological analysis of prostatic adenocarcinoma tissues
Source: Sci Rep. 2022 Jun 21;12:10399. doi: 10.1038/s41598-022-14511-4 (PMC9213544; doi:10.1038/s41598-022-14511-4)
Supplement: Supplementary file 1 — Supplementary Tables. [file 41598_2022_14511_MOESM1_ESM.docx]

**Supplement Table** **1: Characterization of EBV latency profile in EBV-positive prostate carcinoma tissues.** The latency profile was characterized based on the expression of the following latency-associated genes: *EBNA-3B, EBNA-3A, EBNA-2, EBNA-1, LMP-2A, LMP-2, LMP-1, EBNA-LP, EBNA-3C, EBNA-2B, EBER-2, EBER-1, BZLF-1, and BHRF-1.* The table shows mean Ct values for each EBV gene in each sample. ‘No’ indicates an absence of gene expression.

| **EBV latency genes** | **EBNA-LP** | **EBNA-1** | **EBNA-2** | **EBNA-3A** | **EBNA-3B** | **EBNA-3C** | **LMP-1** | **LMP-2** | **LMP-2A** | **LMP-2B** | **EBER-1** | **EBER-2** | **BHRF-1** | **BZLF-1** |
| --- | --- | --- | --- | --- | --- | --- | --- | --- | --- | --- | --- | --- | --- | --- |
| **Sample ID** | **Status (Ct value in "RFU" OR "No")** | | | | | | | | | | | | | |
| Prostate_RNA_7 | 32.75 | 35.83 | 28.46 | 35.14 | 34.67 | No | 29.40 | 38.83 | 33.62 | No | 31.50 | 36.07 | 38.29 | 31.11 |
| Prostate_RNA_12 | 32.07 | 30.11 | 29.06 | 38.89 | 37.08 | No | 31.17 | 36.30 | 29.72 | No | 30.86 | 37.46 | 33.29 | 26.77 |
| Prostate_RNA_14 | 34.65 | 32.83 | 36.91 | No |  | No | 35.19 | 36.73 | 35.37 | 35.82 | 35.03 | 38.49 | 36.72 | 28.06 |
| Prostate_RNA_16 | No | 34.93 | 28.11 | No | 39.46 | No | 38.58 | 39.74 | 35.64 | 36.22 | 36.33 | No | No | No |
| Prostate_RNA_17 | 34.15 | 27.57 | 38.64 | 37.49 | No | No | 34.55 | 39.13 | 37.57 | No | 37.16 | 27.71 | 35.67 | 26.83 |
| Prostate_RNA_19 | No | 36.01 | 33.65 | No | No | No | No | 36.84 | No | 35.33 | 38.17 | No | No | No |
| Prostate_RNA_21 | 34.24 | 36.31 | 26.21 | 36.23 | 39.64 | No | 39.34 | No | 33.29 | 31.22 | 36.25 | No | 38.17 | No |
| Prostate_RNA_22 | No | 35.52 | 35.36 | No | No | No | No | No | 37.58 | 38.64 | 39.8 | No | No | 35.94 |
| Prostate_RNA_24 | 33.88 | 30.55 | 29.37 | No | 37.26 | 32.01 | 32.27 | 35.77 | 29.70 | 32.60 | 30.23 | 32.32 | 32.99 | 27.99 |
| Prostate_RNA_27 | 33.58 | 27.35 | 32.16 | 39.61 | 36.44 | 32.46 | 34.13 | 34.23 | 28.62 | 33.99 | 30.68 | 38.59 | 32.79 | 26.66 |
| Prostate_RNA_28 | No | 34.47 | 34.75 | 39.58 | 37.65 | No | 39.22 | No | 35.38 | No | 35.88 | No | No | No |
| Prostate_RNA_34 | 35.91 | 35.29 | 31.85 | 38.69 | 38.27 | No | 37.85 | No | 38.20 | No | 36.92 | 38.97 | 38.93 | No |
| Prostate_RNA_38 | 33.58 | 29.58 | 31.97 | 39.31 | No | 32.27 | 33.88 | 37.48 | 34.46 | 31.69 | 36.03 | No | 37.47 | 32.93 |
| Prostate_RNA_40 | No | 28.78 | 34.78 | No | No | No | 34.11 | No | 35.91 | 37.60 | 35.6 | 25.68 | 37.31 | 30.84 |
| Prostate_RNA_41 | No | 28.84 | 30.59 | No | 37.74 | No | 31.74 | 33.27 | 27.98 | 38.43 | 29.77 | 32.16 | 31.71 | 26.77 |
| Prostate_RNA_42 | No | No | 32.46 | No | 37.72 | No | 33.12 | 34.22 | 31.78 | 35.25 | 35.10 | 23.17 | 36.26 | 29.30 |
| Prostate_RNA_43 | 36.82 | 34.8 | 36.70 | No | No | No | 38.55 | 39.48 | 35.58 | 35.29 | 36.71 | No | 36.23 | 33.96 |
| Prostate_RNA_51 | No | 34.2 | 31.20 | No | 38.31 | No | 33.76 | No | 34.97 | 31.65 | 37.25 | 36.94 | No | 31.19 |
| Prostate_RNA_52 | 35.65 | 32.02 | 31.34 | 39.08 | 39.16 | 32.26 | 35.24 | 37.49 | 33.06 | 34.08 | 34.77 | 38.41 | 37.60 | 30.95 |
| Prostate_RNA_53 | No | 31.29 | 32.10 | No | 37.43 | No | 34.50 | 36.63 | 31.82 | 35.79 | 38.73 | 38.12 | 36.80 | 30.28 |
| Prostate_RNA_54 | No | 32.48 | 32.38 | No | 39.72 | No | 33.44 | 37.63 | 34.15 | 35.21 | 38.53 | No | No | 32.26 |
| Prostate_RNA_59 | No | 31.51 | 35.88 | No | No | No | 36.35 | No | 36.48 | 37.88 | 38.48 | No | No | 32.90 |
| Prostate_RNA_63 | 34.89 | 28.08 | 28.96 | 39.03 | 35.25 | 32.26 | 32.27 | 36.28 | 31.78 | 37.77 | 32.49 | 38.85 | 35.97 | 27.50 |
| Prostate_RNA_64 | No | 28.39 | 32.37 | No | 39.54 | No | 33.50 | 38.01 | 34.99 | 37.85 | 33.76 | No | No | 30.19 |
| Prostate_RNA_ 65 | 34.72 | 31.28 | 33.27 | No | 38.96 | No | 32.92 | No | No | No | 38.12 | No | No | 39.69 |
| Prostate_RNA_66 | 34.31 | 32.38 | 33.15 | No | No | 31.37 | 36.12 | No | 36.03 | No | No | 26.21 | No | 34.07 |
| Prostate_RNA_69 | 35.92 | 29.00 | 32.42 | 38.77 | 37.89 | No | 35.17 | 37.34 | 34.96 | No | 37.35 | 37.97 | 38.34 | 33.36 |
| Prostate_RNA_72 | 33.28 | 33.20 | 34.54 | No | No | No | 37.07 | No | 36.73 | No | 37.39 | No | 38.32 | 35.05 |
| Prostate_RNA_77 | 36.86 | 28.55 | 29.61 | 35.89 | 35.16 | 32.18 | 32.00 | 37.87 | 34.23 | 35.08 | 35.44 | 35.60 | 36.35 | 27.76 |
| Prostate_RNA_79 | 36.09 | 30.16 | 31.08 | 39.51 | 38.09 | No | 32.52 | No | 32.59 | 34.21 | 37.89 | 35.98 | No | 29.57 |
| Prostate_RNA_82 | No | 37.13 | 34.02 | No | No | No | 37.33 | No | 39.71 | 37.92 | No | 24.83 | No | 36.68 |
| Prostate_RNA_86 | 36.07 | 28.17 | 32.48 | 38.95 | 38.03 | 33.28 | 34.98 | 38.63 | 33.52 | 37.73 | No | 39.28 | 38.90 | 24.54 |
| Prostate_RNA_89 | 34.28 | 29.75 | 32.31 | 38.21 | 38.61 | 32.55 | 33.42 | 38.37 | 32.00 | 38.21 | 35.40 | 38.64 | 36.10 | 31.03 |
| Prostate_RNA_90 | No | 30.59 | 33.73 | 39.87 | 38.19 | No | 34.18 | No | 35.30 | No | 35.94 | 27.19 | 38.43 | 31.13 |
| Prostate_RNA_99 | No | 27.49 | 34.53 | No | 37.33 | No | 35.67 | 39.36 | 38.44 | 37.77 | 36.39 | 30.99 | No | 28.18 |
| Prostate_RNA_100 | No | 28.17 | 37.12 | No | 38.79 | No | 36.35 | 36.81 | 37.92 | 35.48 | 35.86 | 34.85 | 37.85 | 27.16 |
| Prostate_RNA_102 | 35.67 | 28.83 | No | 39.59 | No | No | 36.02 | 35.06 | 39.54 | 35.27 | 35.62 | 36.87 | 36.57 | 31.32 |
| Prostate_RNA_106 | 33.85 | 27.59 | 36.91 | 38.81 | No | 33.14 | 35.53 | 37.41 | 37.52 | 37.15 | 37.79 | No | 35.61 | 28.65 |
| Prostate_RNA_109 | No | 27.36 | No | No | No | No | 37.68 | No | No | No | 35.17 | No | 35.32 | 27.42 |

**Supplement Table** **2: Characterization of EBV latency profile in EBV-positive BPH samples.** The latency profile was characterized based on the expression of the following latency-associated genes: *EBNA-3B, EBNA-3A, EBNA-2, EBNA-1, LMP-2A, LMP-2, LMP-1, EBNA-LP, EBNA-3C, EBNA-2B, EBER-2, EBER-1, BZLF-1, and BHRF-1.* The table shows mean Ct values for each EBV gene in each sample. ‘No’ indicates an absence of gene expression.

| **EBV latency genes** | **EBNA-LP** | **EBNA-1** | **EBNA-2** | **EBNA-3A** | **EBNA-3B** | **EBNA-3C** | **LMP-1** | **LMP-2** | **LMP-2A** | **LMP-2B** | **EBER-1** | **EBER-2** | **BHRF-1** | **BZLF-1** |
| --- | --- | --- | --- | --- | --- | --- | --- | --- | --- | --- | --- | --- | --- | --- |
| **Sample ID** |  | **Status (Ct value in "RFU" OR "No")** | | | | | | | | | | | | |
| **BPH_03** | 38.16 | 27.4 | 23.1 | No | No | No | 33.3 | 33.2 | 29.1 | 21.1 | 30.5 | 34.6 | No | 32.6 |
| **BPH_05** | No | 25.4 | 22.8 | No | No | No | 31.7 | 32.5 | 27.6 | 20.7 | 29.2 | 33.3 | No | 36.1 |
| **BPH_14** | 35.38 | 25.4 | 21.3 | No | No | No | 30.7 | 33.2 | 28.2 | 22.2 | 26.7 | 30.5 | No | 34.6 |
| **BPH_16** | 34.09 | 25.9 | 23.6 | 32.7 | 36.2 | 30.4 | No | 30.2 | 29.5 | 19.5 | 30.4 | 33.3 | 34.7 | 36.0 |
| **BPH_20** | No | 25.7 | 38.2 | No | No | No | 31.6 | 31.0 | 28.9 | 23.2 | 29.3 | 33.1 | No | 35.4 |
| **BPH_21** | 36.03 | 26.1 | 23.5 | No | No | No | 31.8 | 31.3 | 29.4 | 22.0 | 27.1 | 33.6 | 35.6 | 33.2 |
| **BPH_23** | 35.03 | 25.3 | 23.3 | No | No | No | 31.1 | 34.4 | 28.4 | 23.2 | 27.6 | 32.3 | No | 38.9 |
| **BPH_28** | 34.81 | 25.0 | 21.9 | No | No | No | 33.3 | 33.3 | 28.4 | 29.3 | 24.4 | 30.3 | No | 32.2 |
